# Environmental epigenetics and climate change

**DOI:** 10.1093/eep/dvac028

**Published:** 2022-12-14

**Authors:** Michael K Skinner

**Affiliations:** Center for Reproductive Biology, School of Biological Sciences, Washington State University, Pullman, WA 99164-4236, USA

Epigenetics co-evolved with deoxyribonucleic acid sequence as the molecular mechanism to regulate gene expression and chromatin structure and provide the conduit for the environment to influence biology and evolution. All organisms respond to environmental factors through an alteration in epigenetic processes to alter gene expression to facilitate the cellular and organism response. Therefore, environmental epigenetics is the key molecular process involved in how the environment impacts biology. This has been shown now in all organisms from plants to humans, as well as microbes to insects. Therefore, in considering how climate change impacts biology of any organism, environmental epigenetics provides the molecular mechanism to alter gene expression, physiology and phenotypic variation of the organism.

Climate change involves alterations in critical environmental factors such as temperature, air quality, food availability, and water quality and availability. Change in these environmental factors has been shown to have dramatic alterations in the epigenetics, physiology, and phenotypes of all organisms examined. Recent climate change impacts on epigenetics to alter physiology and phenotypes are shown in microbes [1], plants [2, 3], coral reefs [4], insects [5], fish [6, 7], birds [8], and mammals [9]. This does not simply involve the direct environmental actions of these climate change factors on the organism exposed during the development but is also seen in subsequent generations following the ancestral exposure through epigenetic transgenerational inheritance [10]. Therefore, the environmental epigenetics mediates the direct exposure phenotypic variation and physiology but also the generational impact associated with adaptation events and evolution [11]. Climate change impacts on all organisms are mediated through environmental epigenetics to influence genome activity to promote the physiological change and phenotypic variation for the organism. Since this is not only involved in the direct exposure impacts but can be inherited to subsequent generations through epigenetic inheritance, the short-term and long-term impacts of climate change involve environmental epigenetics.

As with any environmental factor or exposure that can promote an epigenetic response to alter the cell biology or physiology of an organism, climate change and the various factors from temperature, to water quality, or to nutrition resources act through environmental epigenetics. In considering the more global and biological impacts of climate change on all organisms, environmental epigenetics is the molecular process involved in the climate change responses. Further insights into and understanding of the role of environmental epigenetics in climate change responses are needed.

Due to the central role of environmental epigenetics in response to climate change, the journal *Environmental Epigenetics* will have a Special Issue on “Environmental epigenetics and climate change” in our 2023 volume issue. We are open to any climate change factors and any organism for consideration. The objective is to better understand the actions of climate change on biology and provide insights into the environmental epigenetic mechanisms involved.
